# Effects of Low Concentration of Glyphosate-Based Herbicide on Genotoxic, Oxidative, Inflammatory, and Behavioral Meters in *Danio rerio* (Teleostei and Cyprinidae)

**DOI:** 10.1155/2024/1542152

**Published:** 2024-09-10

**Authors:** Eduardo Bortolon Ribas, Gustavo Colombo Dal-Pont, Ariana Centa, Marcos Otávio Bueno, Ricardo Cervini, Rosana Claudio Silva Ogoshi, Claudriana Locatelli

**Affiliations:** ^1^ Alto Vale do Rio do Peixe University-UNIARP, Caçador, SC, Brazil; ^2^ Laboratory of Translational Research in Health Alto Vale do Rio do Peixe University–UNIARP, Caçador, SC, Brazil

## Abstract

The glyphosate herbicide is a pesticide widely used in the world and can contaminate soil, air, and water. The objective of this work was to evaluate the toxicity of a glyphosate-based herbicide (GBH) in zebrafish (*Danio rerio*). Fish were exposed to different concentrations of GBH (0, 50, 250, and 500 *µ*g/L) for 96 hours. Brain, liver, and blood were collected for biochemical and genotoxicity analyses, and behavioral tests were performed. The results showed that there was a reduction in the activity of the antioxidant enzymes of catalase (CAT) and glutathione-S-transferase (GST) in the liver at all concentrations and at the highest concentration in the brain. There was also a reduction in lipid peroxidation in the liver at all concentrations of glyphosate. There was an increase in micronuclei in the blood at the 500 *µ*g/L concentration. However, the count of nuclear abnormalities showed no differences from the control. Interleukin-1beta (IL-1*β*) generation was inhibited at all concentrations in the liver and at the highest concentration in the brain. No significant differences were found in the behavioral test compared to the control. The results showed that acute exposure to GBH promoted an inflammatory event, which reduced the efficiency of antioxidants, thus producing a disturbance in tissues, mainly in the liver, causing immunosuppression and generating genotoxicity.

## 1. Introduction

The herbicide glyphosate (N-[phosphonomethyl]glycine) is a broad-spectrum, systemic, postemergent, nonselective herbicide widely used to control weeds in agricultural production, mainly in crops with genetically modified organisms [1–3].

Glyphosate has become the most commercialized pesticide [4, 5], mainly due to the development of transgenic crops [6]. It became the sales leader in the global pesticide market, with around 750,000 tons of the compound used annually and an increase estimated to 920,000 tons by the year 2025 [7].

The intensive use of glyphosate in human activities ends up contaminating the soil, air, and water, in addition to causing changes at different ecological levels, such as in aquatic ecosystems, which can compromise the health of organisms and expose the entire population that uses water to a risk [8].

Recent studies show that exposure to glyphosate has several impacts on environmental health, especially on fish, as they have the ability to absorb and concentrate toxins [9]. A recently published systematic review and meta-analysis study showed that glyphosate affects different developmental stages of zebrafish [10]. Embryons exposed to glyphosate show increased mortality, in addition to an increase in morphological abnormalities such as yolk sac and pericardial edema, associated with body malformations [9, 11]. In adult zebrafish, there was an increase in reactive oxygen species besides memory impairment and a reduction in aggressive behavior, in addition to increased anxiety [11].

Possessing about 70% genetic similarity with humans, the zebrafish is currently an animal model widely used to study the molecular bases of neurobiology, mechanisms involved in the formation of neural circuits, and identification of genes involved in neurological disorders [12]. Furthermore, it has been used in genetic and pharmacological studies to increase the understanding of neurobiological mechanisms [13–15].

Both the intensive use of pesticides in agricultural areas and the improper application by individuals without training or without the necessary instructions for the correct handling of chemical compounds can contaminate aquatic environments and cause adverse effects on organisms [4, 16].

To assess the oxidative potential of the action of a xenobiotic in an organism, oxidative stress biomarkers are used to detect an excessive increase in ROS (oxidative markers) or a reduction in the ability of organisms to combat these antioxidative markers [17].

Oxidative markers are quantifiable through thiobarbituric acid reactive substances (TBARS), which quantify the formation of malondialdehyde (MDA), which is one of the end products of lipid peroxidation (LPO), while antioxidant markers are observed during the activation or immunity of enzymes such as CAT and GST [8, 18].

In addition to oxidative stress, inflammatory processes, which are generated as a response by the immune system, when deregulated, occur to generate tissue damage and are responsible for causing the registration and progression of carcinogenesis in various gains [6, 19, 20].

The immune system is the first line of defense against pathogens or xenobiotics in fish and responds quickly against environmental pressures in the aquatic ecosystem, generating a response from signals such as cytokines, interferons, and tumor necrosis factors, which are formed from an inflammatory reaction [21].

Signaling signals such as cytokines act in different phases of the immune response [22]. At the onset of the inflammatory process, IL-1*β* recruits leukocytes and other cells from the bloodstream into the inflamed tissue.

Given the above, the aim of this research was to investigate the effects of GBH on zebrafish exposed to concentrations of this in terms of behavior, inflammatory, oxidative, and genotoxic processes. Since zebrafish is a vertebrate model widely used in human neurobehavioral studies, the results presented here are relevant not only for the environmental risk assessment, but also for understanding the risk of chronic exposures to low doses of glyphosate.

## 2. Materials and Methods

### 2.1. Animals and Maintenance

Adult specimens of *Danio rerio* (3-4 cm), of both sexes, were obtained from a local commercial supplier and transported to the laboratory of translational research in health at the Universidade Alto Vale do Rio do Peixe (UNIARP) for acclimatization.

The animals were acclimatized over a seven-day period, with 120 specimens distributed across 12 aquariums, each holding 2 liters of dechlorinated water (at a density of 5 fish per liter). The aquaria were maintained under constant aeration, at a temperature of 25°C (±1°C), with a pH of 6.8, nitrite levels at 0.5 ppm, and a 14-hour light/12-hour dark photoperiod. Waste and residue were removed every 2 days, and one-third of the water was replaced regularly. These conditions were optimized for the proper maintenance and welfare of the specimens [23].

Water quality parameters such as pH, temperature, and nitrite were monitored daily and fish were fed twice a day with commercial fish feed. All procedures were approved by the Ethics Committee for the Use of Animals (CEUA-UNIARP), protocol number 001/2021.

### 2.2. Exposure to Glyphosate

After acclimatization, the animals were exposed to concentrations of 0 (control), 50, 250, and 500 *µ*g/L of GBH (Shadow®) for 96 hours of exposure. The product was placed once and the water was maintained without changing during the exposure period. The GBH solution was not renewed as studies show that glyphosate has a long half-life, ranging from 49 to 70 days in water [24].

The concentrations chosen were based on the classification of water bodies by the National Council for the Environment of Brazil (CONAMA) in Resolution no. 357/05, which regulates the maximum permitted concentration of various substances, including glyphosate; in that, in class 1 and 2 waters, fresh water for human consumption and for the protection of aquatic communities, the maximum allowed is 65 *µ*g/L and in class 3 waters, fresh water for human consumption after conventional or advanced treatment, the maximum allowed is 280 *µ*g/L.

The individuals were submitted to euthanasia by cooling, and the caudal peduncle was cut with the aid of a scalpel for blood collection and placed on a slide for later visualization of the erythrocytes. For the other analyses, the liver and the brain of each individual were collected with suitable surgical material (scalpel, scissors, and stainless steel tweezers), deposited in microcentrifuge tubes, homogenized with lysis buffer (50 mM Tris-HCl, pH 7.0; 5 mM EDTA; 140 mM NaCl; 1% Triton X-100, and 0.5% protease inhibitor cocktail (Sigma-Aldrich)), centrifuged at 12000 g for 4 minutes, removed the supernatant, stored in a microcentrifuge tube, and conditioned in a refrigerator at −80°C to the TBARS, CAT, GST, and IL-1*β* tests.

### 2.3. Quantification of Protein

Protein concentration was determined according to the method previously described by Bradford [25]. This method consists of manufacturing total proteins using the Coomassie brilliant blue G-250 dye. Thus, 20 *μ*l of the sample was mixed with 1000 *μ*l of Bradford reagent. The mixture was incubated at room temperature for 3 minutes and the absorbance was read at 595 nm. The analytical curve was prepared in test tubes with successive dilutions of a bovine serum albumin (BSA) (Sigma-Aldrich) solution in a range of 0–50 *µ*g mL^−1^.

### 2.4. Biochemical Analysis of the Antioxidant Profile

#### 2.4.1. Determination of Lipid Peroxidation

It was measured by the absorption of substances that react with thiobarbituric acid (TBARS), using a spectrophotometer in the absorption range of 535 nm [26]. In the assay, homogenates of each sample (100 *µ*L) were mixed with 1 mL of thiobarbituric acid reactive substances (TBARS) and were measured spectrophotometrically. The samples were heated at 100°C for 30 min, and after the tubes were cooled immediately on ice for 10 minutes, they were centrifuged for 10 minutes at 1600 × g at 4°C. The upper clear solution was loaded on 96-well clear plates and the color was measured at 535 nm against the reagent blank using a FLUOstar Omega-Microplate reader (BMG Labtech). The results were expressed in *µ*mol/mg of protein.

#### 2.4.2. Catalase Activity (CAT)

Catalase activity (CAT) was determined by adding a solution of hydrogen peroxide (H_2_O_2_, 10 mM) to phosphate buffer (50 mM, pH 7.0). Catalase present in the sample degrades hydrogen peroxide [27]. In a quartz cuvette, 2 mL of hydrogen peroxide solution (10 mM) in phosphate buffer (50 mM, pH 7.0) was added to 10 *µ*L of the sample. Reading was performed every 10 seconds for 40 seconds, evaluating the absorbance decay at 240 nm. The results were expressed in mmol/mg of protein.

#### 2.4.3. Glutathione-S-Transferase (GST)

The glutathione-S-transferase (GST) was determined using the method by HABIG et al. [28]. The GST present in the sample converts CDNB (1-chloro-2,4 dinitrobenzene) into TNB (trinitrobenzene) through conjugation with the GSH present in the reaction medium. In a quartz cuvette, 0.98 mL of phosphate buffer (0.1 M, pH 7.0), 10 *µ*L of CDNB (0.1 M), and 10 *µ*L of reduced glutathione (0.1 M GSH) were added to 10 *µ*g of the sample. The first reading was followed and after 1 minute, the second reading was taken in the absorbance range of 340 nm. The results were expressed in mmol/mg of protein.

### 2.5. Interleukin Activity (IL-1*β*)

To quantify IL-1*β* in zebrafish homogenates, instructions were followed according to the manual for the Elisa kit from the manufacturer Cusabio Technology LLC®.

The assay employs a sandwich immunoassay technique, in which IL-1*β* is fixed onto the plate along with the standard and sample. They are pipetted into the wells causing the IL-1*β* to bind to the immobilized antibody. After removing unbound substances, a specific antibody conjugated with biotin (100 *µ*L) is added to the wells, washed (200 *µ*L), and an avidin peroxidase conjugate (HRP-avidin) (100 *µ*L) is added. Another wash (200 *µ*L) is performed to remove any unbound enzyme-avidin reagent and then a substrate (100 *µ*L) is added to the wells and a hue proportional to the amount of IL-1*β* present in the sample is observed. Absorbance is measured at 450 nm. Values were expressed in pg/mg of protein.

### 2.6. Micronucleus Test and Nuclear Abnormalities

Blood smear slides were fixed with methanol and stained with 20% Giemsa. 1000 cells were counted on each slide under an optical microscope with 1000x magnification, totaling 6 slides per treatment (control, 50 *µ*g/L, 250 *µ*g/L, and 500 *µ*g/L). In the counts, cells were considered normal when the oval-rounded shape characteristic of the species was maintained; the micronucleus was characterized as a small intracytoplasmic body, resembling the nucleus in color, varying from 1/3 to 1/20 in size with the nucleus and separated from it without a binding bridge [29]. Nuclear abnormalities were grouped and summed considering all nuclei that presented invaginations (blebbed, lobed, and notched); binucleated, when the erythrocyte presented two apparent nuclei; vacuolated, when the nucleus presented a cavity within its nuclear limit [30]; and karyorrhexis, when the erythrocyte presents a nucleus decomposed into several micronuclei in the cytoplasm [31].

### 2.7. Behavioral Test

#### 2.7.1. Light/Dark Test

**Figure 1:**
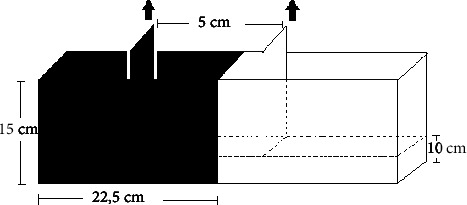
Light/dark test tank. The triangle consists of a rectangle, with a light and a dark side and with a central compartment with removable doors with the dimensions described below. Fonte: Stewart et al. [15].

For this test, a rectangular glass aquarium (45 cm long × 10 cm wide × 15 cm high) was used, half transparent and the other dark opaque in order to be careful not to reflect the image of the animal (Figure 1), filled with dechlorinated water to a height of 10 cm, totaling 4.5 liters of water. The apparatus has a central compartment with removable doors, each one in the color of the side on which it is located, with 5 cm of distance between them.

The animals were transferred to the behavior analysis aquarium after 96 hours of exposure to GHB. The behavior was evaluated by a trained observer and recorded in spreadsheets for subsequent data analysis.

Each animal (*n* = 10) was transferred from its home aquarium with the aid of a net (5 × 5 cm), and placed in a beaker containing its water, always by the same researcher. The specimen was transported to the room where the test aquarium was previously arranged, prepared, and lifted with the aid of a net (5 × 5 cm) into the central compartment. After an interval of 5 minutes for the individual's acclimatization, the following exploratory observations were started: latency time in which the animal remained in the central compartment after removing the removable doors, number of alternations between light and dark sides and vice versa, and total time spent on the light side and total time spent on the dark side. Each animal was evaluated for 15 minutes with the water in the apparatus changed after every 3 specimens [32].

### 2.8. Statistical Analysis

For statistical analysis and graphs, the program used was GraphPad® Prism 9.3.1. When the data tended to normality, the one-way ANOVA test was used followed by the Bonferroni test for multiple comparisons. When it was assumed that the data were nonparametric, the Kruskal–Wallis test was used followed by the Dunn's test for multiple comparisons. Asterisks indicate statistical differences compared with the control group: ^∗^(*p* < 0.05), ^∗∗^(*p* < 0.01), ^∗∗∗^(*p* < 0.001), and ^∗∗∗∗^(*p* < 0.0001). Data were reported as mean ± standard deviation.

## 3. Results

**Figure 2:**
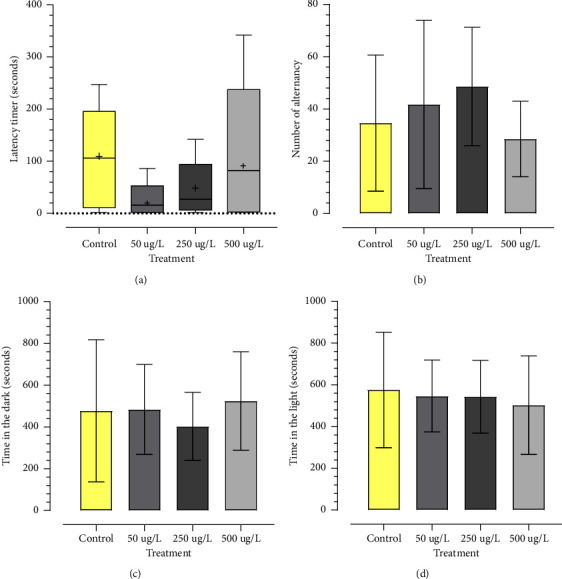
Effects of GBH exposure (0, 50, 250, 500 *μ*g/L) on groups of adult zebrafish (n = 10) in the 15-minute light/dark behavioral test (900 seconds); zebrafish was exposed to GBH for 96 hours. Data analyzed by one-way ANOVA, followed by post hoc Kruskal–Wallis when the standard deviation (SD) showed a significant value in the Brown–Forsythe test. Values expressed as mean ± standard deviation in column graphs. Average (+) on box plot charts.

Figure 2 shows the exposure effects of adult zebrafish exposed to glyphosate-based herbicide (GBH) through the light/dark environment test. No significant differences (*p* < 0.05) were found in the behavior of animals exposed to glyphosate concentrations when compared to the control group in none and light time (Figure 2).

**Figure 3:**
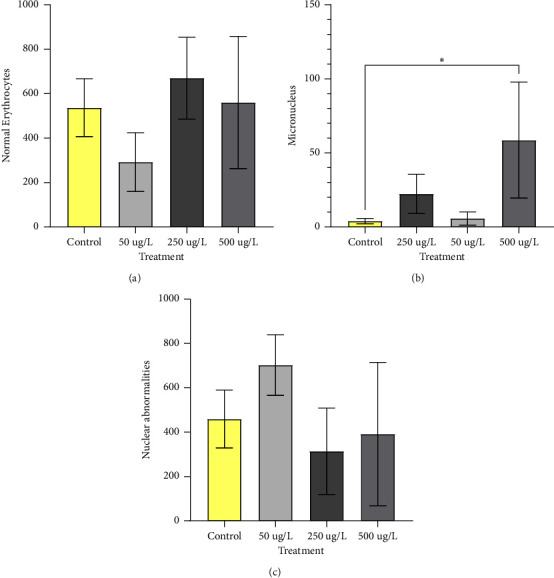
Frequencies of MN, AN and normal erythrocytes observed in a thousand cells per slide in adult zebrafish exposed to GBH. Effects of glyphosate exposure (0, 50, 250, 500 *μ*g/L) in groups of adult zebrafish (n = 8). Zebrafish were exposed to GBH for 96 hours. Data analyzed by one-way ANOVA. Values expressed as mean ± standard deviation. Where ^∗^(*p* < 0.05).

**Figure 4:**
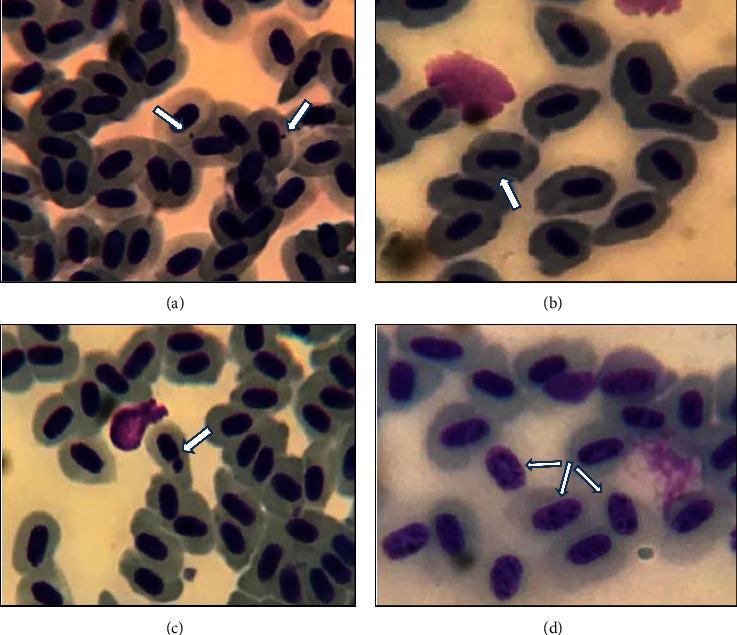
Danio rerio erythrocytes. Smear containing D. rerio erythrocytes stained with 20% giemsa. (a) micronucleus (in the two arrows), with surrounding normal erythrocytes. (b) Nuclear invagination (arrow) of the blebbed type. (c) micronucleus (arrow). (d) karyorrhexis (on the three arrows).

Frequencies of MN, AN, and normal erythrocytes in zebrafish are shown in Figure 3. As observed in the graphs of the statistical analysis, only the frequency of micronuclei (Figure 3) with the highest concentration showed a significant difference (*p* < 0.05) compared to the control group. Concentrations lower than 250 *µ*g/L and 50 *µ*g/L did not show significance. Figures 4(a) and 4(c) shows the presence of micronuclei in the slides analyzed.

The frequencies of normal and NA erythrocytes (Figure 3) did not show statistical differences in any of the treatments compared to the control group. Examples of erythrocytes containing NA are shown in Figures 4(b) and 4(d).

**Figure 5:**
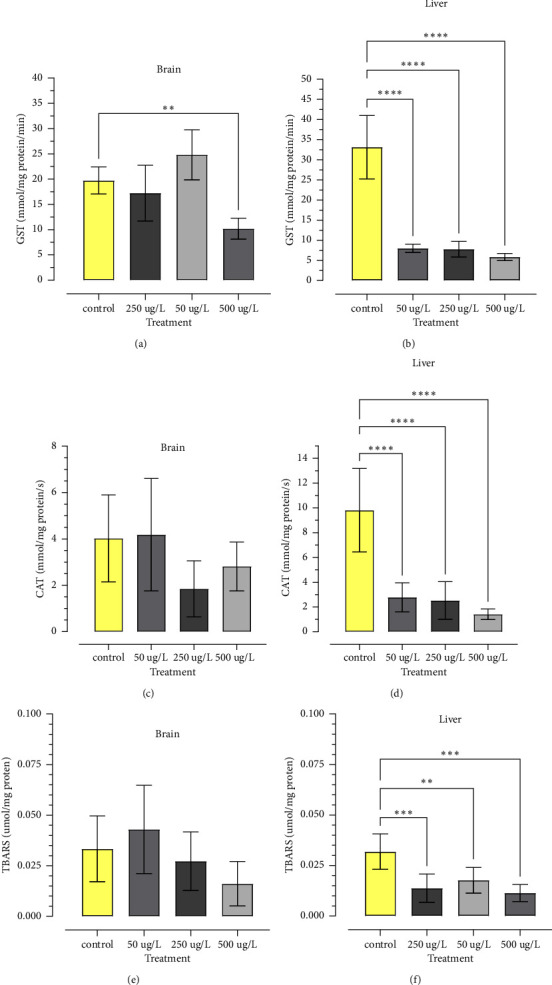
CAT and GST enzyme activity and TBARS levels in zebrafish brain and liver (n = 10) exposed to concentrations (0, 50, 250, 500 *μ*g/L) of GBH for 96 h. Data analyzed by one-way ANOVA. Values expressed as mean ± standard deviation. Where ^∗^(*p* < 0.05), ^∗∗^(*p* < 0.01), ^∗∗∗^(*p* < 0.001), ^∗∗∗∗^(*p* < 0.0001).

The activity of the GST enzyme in the brain showed a reduction in the group of zebrafish exposed to 500 *µ*g/L (*p* < 0.01) of GBH in relation to the control (Figure 5(a)); in the liver, it showed a reduction in the treatments of 50 *µ*g/L, 250 *µ*g/L, and 500 *µ*g/L (*p* < 0.0001) of GBH in relation to the control (Figure 5(b)).

The activity of the CAT enzyme in the brain did not show a significant result in any of the groups of zebrafish exposed to GBH in relation to the control (Figure 5(c)), but it showed a significant difference in the liver, with a reduction of enzymatic activity in the groups exposed to 50 *µ*g/L, 250 *µ*g/L, and 500 *µ*g/L (*p* < 0.0001) of glyphosate when compared to the control treatment (Figure 5(d)).

The determination of lipid peroxidation through TBARS did not show significant results in the brain of zebrafish exposed to GBH in any of the treatments compared to the control (Figure 5(e)), and as for the liver, it showed a reduction in the treatment of 50 *µ*g/L, with a significance of *p* < 0.01, when compared to the control and a reduction in the treatments of 250 *µ*g/L and 500 *µ*g/L, with a significance of *p* < 0.001, compared to the control (Figure 5(f)).

**Figure 6:**
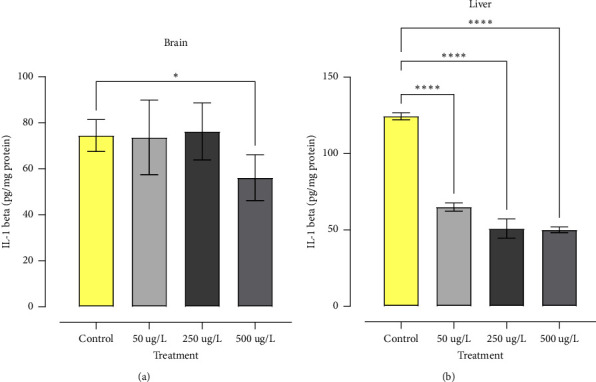
IL-1*β* levels in brain and liver of zebrafish exposed to concentrations (0, 50, 250, 500 *μ*g/L) of GBH for 96 h. Data analyzed by one-way ANOVA. Values expressed as mean ± standard deviation. Where ^∗^(*p* < 0.05), ^∗∗^(*p* < 0.01), ^∗∗∗^(*p* < 0.001), ^∗∗∗∗^(*p* < 0.0001).

IL-1*β* levels in zebrafish's brain and liver are shown in Figure 6. Brain IL-1*β* level decreased in the 500 *µ*g/L treatment compared to the control (*p* < 0.01) (Figure 6(a)). In the liver, IL-1*β* levels showed a reduction in all treatments when compared to the control (*p* < 0.0001) (Figure 6(b)).

## 4. Discussion

The results showed that acute exposure to GBH promoted an inflammatory event, which reduced the efficiency of antioxidants, thus producing a disturbance in tissues, mainly in the liver, causing immunosuppression and generating genotoxicity.

In the present study, a slightly longer average time was observed on the light side than on the dark side, indicating a reduced anxiety behavior in general in all treatments, but with no significant difference with the control. One way of observing zebrafish behavioral actions is the light/dark environment test [33]. Scototaxis, the preference for dark environments, is a typical behavioral strategy observed in zebrafish. This behavior reflects an evolutionary adaptation to avoid potential predators, as seeking darker areas offers camouflage and reduces exposure to threats in their natural habitat [34].

Studies show that glyphosate caused behavioral changes in zebrafish, such as the study by Chaulet et al. [35], which indicated the addition of the anxiolytic characteristic of the animal exposed to 3 and 5 mg/L of a glyphosate-based herbicide. The same anxiolytic behavior was noted in the study by Maximino et al. [33], in which groups of zebrafish showed behavioral changes in treatments of 0.065 mg/L and 0.5 mg/L with Roundup® and 0.5 mg/L with glyphosate.

In other animals, behavioral effects were also observed when exposed to glyphosate or its commercial compounds, such as fish [36, 37], rats [38], and insects [39].

In all the treatments in which the animals were exposed to GBH, in the present study, they did not promote possible significant changes in the neurological status of the zebrafish according to the behavioral test evaluated, being necessary further studies for a better evaluation of the toxicity of glyphosate as a pollutant of aquatic ecosystems and a possible behavioral change in fish populations. The results are possibly directly related to the type of formulation used, as Bridi et al. [11] observed such a change in treatment with Roundup®.

Biomarkers have been widely used as tools to detect exposure and assess the effects of genotoxic pollution on ecosystems and consist of tests that assess chromosomal anomalies, DNA breaks, frequency of MN, and other AN, such as the micronucleus test [40]. Among the pollutants that are potentially genotoxic are pesticides [40], including organophosphates, as the study by Nahas et al. [41] who observed an increase in micronuclei in a group of fish *Oreochromis niloticus* exposed to pesticides.

The genotoxic potential was found in the glyphosate herbicide, as reported by Cavalcante et al. [42] with the MN test in groups of fish *Prochilodus lineatus* exposed to Roundup®, as well as Qin et al. [43] demonstrated that glyphosate exposure for 24 hours in cells from the fish *Misgurnus anguillicaudatus* had a significant increase in MN at a dose of 400 mg/L when compared to the control group, and AN at a dose of 560 mg/L compared to the control group.

The occurrence of micronuclei by endogenous origin has been reported by several authors and shows a great variability between species, with values between 0 and 13 MN per 1000 cells [44–46]. According to Canedo et al. [47], in reviewed studies on the genotoxic potential of glyphosate, the basic level of MN found in zebrafish erythrocytes was 0% and the maximum level was 2.57%.

In the present study, a significant difference was found in the frequency of micronuclei in the 500 *µ*g/L treatment compared to the control group (mean of 6%), demonstrating a disruption in the cell's chromosomal system caused by GBH. The 50 *µ*g/L and 250 *µ*g/L treatments did not differ statistically from the control group. There was no significant difference in the frequency of AN and in the amount of normal erythrocytes, indicating that the GBH concentrations used in a 96-hour exposure to zebrafish peripheral erythrocytes did not cause AN when compared to the control group.

Studies with glyphosate demonstrate the generation of MN and AN, such as that of Çavas and Könen [48], who found an increase in the frequency of MN and AN in *Carassius auratus* erythrocytes exposed to glyphosate at concentrations of 5, 10, and 15 mg/L for periods of 48, 96 and 144 hours. Caramello and Jorge [47] found an increase in MN and AN in *Prochilodus lineatus* erythrocytes exposed to different concentrations of Roundup©.

Similar results were found by Odetti et al. [49] in *Caiman latirostris hatchlings* where the study showed that although glyphosate-based formulations can promote an increase in the frequency of micronuclei even when used in low concentrations, this effect does not is related to oxidative stress. However, deleterious genetic effects on fish and alligator species can be a threat to survival and health status.

Oxidative stress results from a mismatch between the production of ROS and the antioxidant capacity resulting from endogenous agents or environmental factors leading to harmful actions of ROS on cells [6]. The antioxidant defense system is a mechanism present in organisms to prevent the damage that ROS can cause and includes enzymes such as CAT and GST [50].

The CAT enzyme is a primary antioxidant defense component that contains iron and facilitates the removal of H_2_O_2_, which is metabolized to molecular oxygen and water in the metabolization of xenobiotics [8], and GST catalyzes glutathione processes in the process of biotransformation of xenobiotics [51].

Any change in antioxidant enzyme activity is indicative of oxidative activity [52]. Both the increase and depletion in the activity of these enzymes when exposed to pollutants suggest an activity of these substances in organisms [53]. The reduction of antioxidant capacity ends up making the fight against the activity of oxidizing substances in the body inefficient [19].

In this study, there was a significant decrease in GST activity in the 500 *µ*g/L treatment and no significant CAT activity in the zebrafish brain, however, in the liver there was a significant depletion of GST and CAT in all glyphosate treatments with the zebrafish.

In a study by Lushchak et al. [54], a significant decrease in catalase (CAT) activity was observed in Carassius auratus exposed to concentrations exceeding 10 mg/L of Roundup® for 96 hours. Similarly, in Rhamdia quelen (silver catfish), CAT activity was reduced after exposure to 1.21 mg/L of Roundup® for 96 hours [55]. In Prochilodus lineatus, exposure to 10 mg/L of Roundup® for 96 hours resulted in increased glutathione S-transferase (GST) and lipid peroxidation (LPO) activity in the liver, while the activities of superoxide dismutase (SOD) and glutathione peroxidase (GPx) were reduced [56]. Additionally, Gallegos et al. [58] reported a decrease in CAT activity in the brain of rats exposed to glyphosate, and Zancanaro et al. [58] observed reduced CAT activity in the liver and kidney tissues of rats exposed to glyphosate or a glyphosate-based herbicide (GBH).

The liver is the main source of GST and CAT in fish, in contrast to the activity in the brain, which is generally low [59, 60], reinforcing the analysis of the present study, in that there was no significant activity of CAT in all treatments and of GST at concentrations of 50 *µ*g/L and 250 *µ*g/L in the brain of zebrafish exposed to glyphosate for 96 h.

As a response to oxidative damage to biomolecules, LPO was analyzed using the TBARS test, in which LPO damage was caused by the generation of ROS or changes in antioxidant capacity [51]. In analyzes carried out with TBARS, no significant changes were found in the brain, and there was a decrease in levels in the liver and in all treatments with zebrafish.

In the study by Moura et al. [61], there was a significant increase in TBARS levels in liver (6 h and 48 h) and muscle tissue (24 h) in the hybrid fish jundiara (*Leiarius marmoratus* x *Pseudoplatystoma reticulatum*) after exposition of Roundup©, although Glusczak et al. [62] observed glyphosate depletion in Jundiá (*Rhamdia quelen*) exposed to 0.2 mg/L and 0.4 mg/L of glyphosate for 96 hours.

Some studies have reported an increase in brain LPO levels in zebrafish [9, 63] and in Jundiá [64], and no changes in levels in Pintado da Amazônia [65].

It is important to highlight that different LPO responses are dependent on the fish species and the specific characteristics of tissues with different levels of peroxide production and also on variations in antioxidant responses [51]. The reduction in LPO can be explained by the activity of other enzymes that were not evaluated in the study, such as superoxide dismutase, glutathione peroxidase, and reduced glutathione (GSH).

The increase in the frequency of micronuclei observed in the present study may also be related to a decrease in the activity of CAT and GST enzymes, since damage to cellular DNA can cause a change in the transcription of proteins, including antioxidant enzymes. However, further studies evaluating the expression of antioxidant proteins in zebrafish exposed to low concentrations of glyphosate are necessary to confirm these findings. Reduction in catalase activity associated with changes in glutathione gene expression was recently found by Chitolina et al. [66] in the ovaries of rats exposed to low doses of GBH for 28 days.

IL-1*β* is a pro-inflammatory cytokine, which activates cells of the immune system such as macrophages, lymphocytes and monocytes, leading to an inflammatory effect in the face of any environmental stressor, tissue injury, or pathogen invasion [20, 67].

Interleukin-1*β* (IL-1*β*) has been extensively studied across various fish species, demonstrating its pro-inflammatory role in response to xenobiotics [68]. Previous studies have confirmed its activity in American catfish (*Ictalurus punctatus*) [69], common carp (*Cyprinus carpio* L.) [70], and southern bluefin tuna (*Thunnus maccoyii*) [71]. These findings reinforce the critical involvement of IL-1*β* in the immune response triggered by the presence of environmental contaminants.

Glyphosate has been shown to induce the production of pro-inflammatory cytokines in various studies. For example, Pandey, Dhabade, and Kumarasamy [72] reported an increase in IL-1*β* levels in the adipose tissue of rats. Similarly, an elevation of IL-1*β* was observed in the liver of common carp (*Cuprinus carpio*) exposed to 5 mg/L and 50 mg/L of glyphosate over 15, 30, and 45 days [73], as well as in the kidneys of rats [74]. Notably, in Oreochromis niloticus (*Nile tilapia*), glyphosate exposure led to a significant increase in IL-1*β* levels in the gill tissue, further corroborating its role in the inflammatory response to environmental contaminants [75].

In the study by Ma and Li [21], glyphosate caused an increase in IL-1*β* levels in the liver and kidneys of common carp exposed to 52.08 mg/L and 104.15 mg/L for up to 168 hours and a decrease of IL-1*β* levels in the spleen when compared to control.

This study showed an opposite result in the levels of IL-1*β*, resulting in the inhibition of IL-1*β* and demonstrating that glyphosate generated immunosuppression in zebrafish, leaving the animal deficient in a critical cytokine for the onset of the inflammatory process, and this result may be correlated with the increase in MN observed in the treatment with 500 *µ*g/L of GBH. The reduction in IL-1*β* observed in this study may, in turn, be associated with the inhibition of caspase-1, which is responsible for the conversion of pro-IL-1*β* into active IL-1*β*. Recent studies have shown that glyphosate can inhibit caspase pathways, reducing the apoptosis process, which is one of the factors related to the potential tumorigenic effect of glyphosate [76].

Future studies may build upon confirming some data found in the present study, such as evaluating the expression of antioxidant enzymes and assessing caspase-1 to determine if it is inhibited by exposure to GBH and perhaps associated with the reduction in IL-1*β* activity.

## 5. Conclusion

The present study demonstrated an implicit relationship between toxicity mechanisms in the face of acute exposure to GBH. The present study demonstrated a clear relationship between toxicity mechanisms and acute exposure to glyphosate-based herbicides (GBH). GBH exposure led to an imbalance in antioxidant enzyme activity, with a reduction in CAT and GST levels, along with a decrease in TBARS. Additionally, a reduction in the pro-inflammatory cytokine IL-1*β* was observed. These findings highlight a more pronounced deregulation of enzymatic activity in hepatic tissue compared to brain tissue, suggesting a higher inflammatory response in the liver than in the brain.

Similarly, the analysis of zebrafish blood found genotoxicity in the face of exposure to GBH from the generation of micronuclei, and the behavioral analysis did not obtain a significant difference in the face of the evaluated test.

The data presented can enrich studies about the mechanisms of exposure to glyphosate in zebrafish in the face of behavioral, inflammatory, oxidative, and genotoxic analyses, which can provide information about the toxicity of glyphosate in exposed fish. Based on these data, it can be inferred that glyphosate-based herbicides, even at low concentrations and over short exposure periods, can induce damage, as evidenced by the altered activity of various oxidative enzymes.

## Data Availability

The data used to support the findings of this study are available from the corresponding author upon request, and these data are archived on the platform of the institution Universidade Alto Vale do Rio do Peixe associated with the health translational research laboratory.
